# Toxicological Profile of Chlorophenols and Their Derivatives in the Environment: The Public Health Perspective

**DOI:** 10.1155/2013/460215

**Published:** 2013-04-03

**Authors:** Etinosa O. Igbinosa, Emmanuel E. Odjadjare, Vincent N. Chigor, Isoken H. Igbinosa, Alexander O. Emoghene, Fredrick O. Ekhaise, Nicholas O. Igiehon, Omoruyi G. Idemudia

**Affiliations:** ^1^Department of Microbiology, Faculty of Life Sciences, University of Benin, PMB 1154, Benin City 300001, Nigeria; ^2^Department of Biochemistry and Microbiology, University of Fort Hare, Private Bag X1314, Alice 5700, South Africa; ^3^Environmental Engineering & Water Technology Department, UNESCO-IHE Institute for Water Education, Westvest 7, 2611 AX Delft, The Netherlands; ^4^Department of Basic Sciences, Benson Idahosa University, PMB 1100 Benin City 300001, Nigeria; ^5^Department of Microbiology, Faculty of Biological Sciences, University of Nigeria, Nsukka 410001, Nigeria; ^6^Department of Chemistry, University of Fort Hare, Private Bag X1314, Alice 5700, South Africa

## Abstract

Chlorophenol compounds and their derivatives are ubiquitous contaminants in the environment. These compounds are used as intermediates in manufacturing agricultural chemicals, pharmaceuticals, biocides, and dyes. Chlorophenols gets into the environment from a variety of sources such as industrial waste, pesticides, and insecticides, or by degradation of complex chlorinated hydrocarbons. Thermal and chemical degradation of chlorophenols leads to the formation of harmful substances which constitute public health problems. These compounds may cause histopathological alterations, genotoxicity, mutagenicity, and carcinogenicity amongst other abnormalities in humans and animals. Furthermore, the recalcitrant nature of chlorophenolic compounds to degradation constitutes an environmental nuisance, and a good understanding of the fate and transport of these compounds and their derivatives is needed for a clearer view of the associated risks and mechanisms of pathogenicity to humans and animals. This review looks at chlorophenols and their derivatives, explores current research on their effects on public health, and proffers measures for mitigation.

## 1. Introduction

Chemical substances are essential in many economic activities and are a significant part of daily life. They provide society with a wide range of benefits, particularly increased agricultural and industrial productivity and improvements in the control of diseases. Nevertheless, chemical compounds have the potential to cause considerable environmental and health problems from production through to disposal. Xenobiotics are a major cause for concern world over, given their recalcitrance to degradation by artificial or natural means and adverse effects on humans and the ecobiota. Global increase in industrial and agricultural activities has led to the production of new xenobiotics such as chlorophenolic compounds. Chlorophenols are environmental pollutants introduced into the environment as a result of chemical and pharmaceutical industry activities [1–3]. The wide spread presence of these compounds in the environment is also related to the production use and degradation of numerous pesticides, such as chlorobenzenes [4] and chlorinated cyclohexanes [5].

Over the past five decades, chlorophenols have become quantitatively significant pollutants in the environment and their treatment, disposal, and general management have become a serious challenge to stakeholders in the environment and health sectors [6]. In an effort to remedy the effects of xenobiotics like chlorophenols, bioremediation using microorganisms has been suggested [1, 7, 8]. The diversity, versatility, adaptability, and metabolic potentials of a number of microbes have been harnessed and applied in bioremediation of environmental contaminants [7]. However, a number of contaminants have been shown to be unusually refractory to microbial degradation; thus they are either not metabolizable or are transformed into other metabolites that accumulate in the environment [9]. 

The transformation of chlorophenols in particular could lead to increase in toxicity of intermediate compounds or end products due to formation of electrophilic metabolites that may bind and damage DNA or gene products [2]. The noxious influence of chlorophenols and their derivatives on the ecobiota may lead to acute toxicity, histopathological changes, mutagenicity, and cancer. These serious health issues make it imperative not only to control chlorophenols in the environment but also to assess and understand their fate in the environment with a view to protecting the environment and preserving the public health communities. This review addresses the incidence and fate of chlorophenolic compounds in the environment with special emphasis on their adverse effects on the ecobiota.

## 2. Policy Regulations of Chlorophenols in the Environment

**Figure 1:**
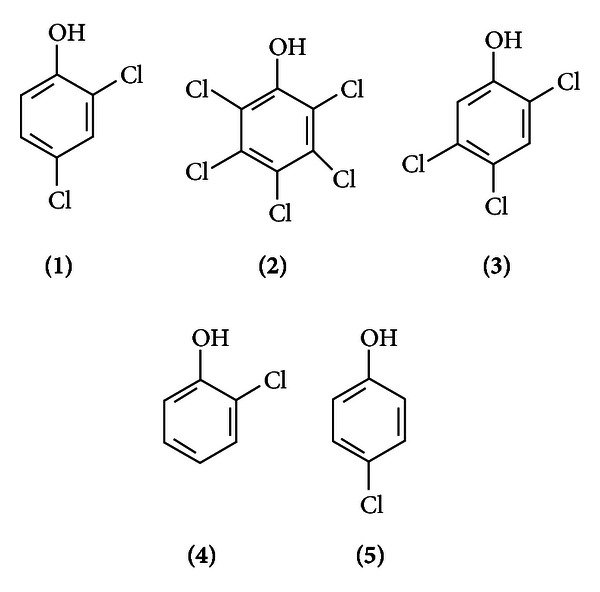
Commercially the most important chlorophenols. 2,4-dicholorophenol (2,4-DCP) **(1)**; pentachlorophenol (PCP) **(2)**; 2,4,5-trichlorophenol (2,4,5-TCP) **(3)**; 2-chlorophenol (2-CP) **(4)** and 4-chlorophenol (4-CP) **(5)**.

The presence of chemicals in the environment due to its use for various purposes affects the quality of air, water, soil, and human health. It is important to assess the risks of these pollutants to the ecosystem in order to create a firm basis for environmental policy formulation. To this end, governmental agencies across the globe have issued several policy statements aimed not only at preserving the health of the teeming world population but also the environment. In 1976, the European Union (EU) categorized 132 dangerous substances (based on their toxicity, stability, and bioaccumulation) that should be monitored in waters [10]. Amongst these substances are organochlorinated compounds (or chlorophenols) and substances that can be converted to organochlorinated compounds (Table 1). The structures of some commercially important chlorophenols are shown in Figure 1.

The European Union made recommendations for establishments to ensure monitoring programs that controls the emission of industrial discharges from textile, refineries, pulp, and paper factories into the air, water, and soil [10–12]. The general strategy for water protection and the prioritized dangerous substances to be controlled includes hexachlorobenzene (HCB), hexachlorocyclohexane (HCH, lindane), polycyclic aromatic hydrocarbons (PAHs), pentachlorobenzene, and pentachlorophenol (PCP) with the aim of protecting the health of the general population [13, 14]. The European Union has set target limits of 0.1 *μ*g/L and 0.5 *μ*g/L as maximum concentrations for pesticide and its product of degradation and for total concentration of pesticides, respectively, in the environment [15]. The term relevant metabolite with respect to toxicity was introduced in the EU Directive 91/414/EEC [16], with subsequent amendments. The legislation concerning placing product in markets and subsequent guidance on the use has been provided [17]. In the USA, the Environmental Protection Agency (EPA) has established the maximum level for each pesticide or its transformation products according to their toxicity [18]. For public protection against toxic effects of pesticides, regulatory agencies in several countries have established standards specifying the acceptable residual levels of each pesticide in various foodstuffs [19]. Similarly, the World Health Organization (WHO) has sets of basic acceptable minimum standards for these toxicants which are evaluated and reviewed periodically. The acceptable daily intakes (ADIs) of pesticides will be seen in the report of the joint meeting of the Food and Agriculture Organization (FAO) panel of experts on pesticide residues in food and the environment and the WHO core assessment group on pesticide residues [20, 21]. 

Two fundamental approaches exist for dealing with environmental contamination as a result of discharged chemicals; the first approach is to prove the safety of a xenobiotic chemical and its potential by-products prior to widespread use and discharge [22, 23]. The second approach is not to use the chemical unless the chemical's toxicity and risk can be clearly determined [22, 23]. In a bid to deal with these toxicants, European countries within the European Union have restricted the use of such chemicals as is found in Table 1, because of issues surrounding their potential environmental toxicity [10, 22]. The United States has taken the second approach and allows the use of xenobiotic compounds like chlorophenols, even though the toxicity and problems associated with exposure to low concentrations of these compounds have not been clearly determined [24]. The problem with this second approach is that the effects of low concentrations of contaminants in the environment can be so complex and difficult to determine that clear scientific proof of toxicity may never be absolutely determined, even though they cause environmental or human harm [24].

## 3. Exposure of the Environment to Chlorophenols

Environmental and occupational exposures to pesticides as a risk factor for hematopoietic tumors have been widely studied mainly among farmers and agricultural workers, in rural communities and in the pesticide manufacturing industries [25, 26]. Occupational exposure to pesticides includes a broad range of occupational categories such as end-users (farmers and applicators) and workers during the manufacturing process (manufacturing workers) both undergoing diverse qualitative and quantitative exposures [26, 27]. 

One of the primary concerns of the environment's exposure to chlorophenols is their potential to contaminate aquatic ecosystems (ground and surface waters) and consequently posing great risk to humans and other organisms associated with the food chain of the aquatic ecobiota [28]. The situation could be made worse by the fact that chlorophenols are so recalcitrant that they may maintain high toxicity levels (unchanged) within the environment for a very long time [8]. Exposure to chlorophenols has been associated with industries that produce textiles, leather products, domestic preservatives, and petrochemical industries [26–28]. Occupational exposures have been observed to occur through inhalation and dermal contact with this compound at workplaces [28]. Workers' exposure was reported in plants producing chlorinated pesticides or fungicides as well as in industrial incinerator, wastes plants, and electrical utility line-men in contact with chlorophenol-treated poles used in electric line construction [28]. Occupational exposure of workers to phenoxy herbicides has been associated with increased morbidity and mortality due to cancer of respiratory system, lymphoma, and myocardial ischaemia [28]. A positive correlation was also shown to exist between non-Hodgkin's lymphoma appearance among children and frequency of pesticide use [29]. The investigations of 10,000 workers employed in vinyl chloride production factories showed that they suffered from liver and lung cancer [30]. 

The International Agency for Research on Cancers categorized chlorophenols into five groups as follows: pentachlorophenol (PCP), 2,3,4,6-tetrachlorophenol (2,3,4,6-TeCP); 2,4,6-trichlorophenol (2,4,6-TCP), 2,4,5-trichlorophenol (2,4,5-TCP) and 2,4-dichlorophenol (2,4-DCP) as belonging to the 2B group of potential human carcinogens [31, 32]. This category encompasses chemical agents for which sufficient evidence of carcinogenicity in animals and inadequate evidence of carcinogenicity in humans have been established. The World Health Organization classified some chlorophenols (2,4,6-trichlorophenol, 2,4,5-trichlorophenol, and pentachlorophenol) as compounds suspected of having carcinogenic properties [31, 33]. 

### 3.1. Effect of Environmental Exposure to 2,4,5-Trichlorophenol and Its Derivatives

Although the application of 2,4,5-trichlorophenol (2,4,5-TCP) as biocide has been restricted in many countries, it is still used as a fungicide in wood and leather impregnation in many parts of the world [34]. Other routes by which 2,4,5-TCP could gain entrance into the environment include article mills where they are used in wood pulp bleaching, and as components of drinking water [35]. The formation of chlorocatechols from chlorinated phenols in mammals has been proven. Dichlorocatechols, including 4,5-dichlorocatechol (4,5-DCC), may be formed from both TCP and PCP in rodents [36]. 4,5-DCC and, similarly, 4,6-dichloroguaiacol (4,6-DCG) are formed in high amounts during article production; thus, their concentrations in sewages and polluted surface water may be very high, >3 mg/L [37]. Chlorinated guaiacols were actively accumulated in aquatic biota, including fish [38], indicating their potential risk to consumers of such fish products. Furthermore, the presence of 4,6-DCG was reported in drinking water [35] and in the air of areas exposed to industrial pollution [39]. The 2,4,5-TCP has also been determined in drinking water [35], as it is formed as a result of water disinfection (chlorination) [2]. 

### 3.2. Incidence and Effect of 2,4,5-Trichlorophenol and Its Derivatives on Living Organisms

As previously highlighted, chlorophenols are known to be harmful toxic substance, because they easily penetrate skin and epithelium, leading to damage and necrosis [2]. It is also known that workers employed in the production of phenoxy herbicides and chlorophenols often suffer from heart disease, asthma, non-Hodgkin's lymphoma, lung cancer, and sarcoma [28]. The exposure of people in Jarvela (Finland) to drinking water contaminated with chlorophenols, including TCP, caused increased incidence of digestive tract infections, asthma, depression, and morbidity [40]. Patients who had high levels of chlorophenols in their blood were reported to have increased interleukin-8 serum levels and T-lymphocyte dysfunction [41]. 

The ubiquity of exposure by the general population to TCP has been proven in some investigations. In a German Environmental Survey [42], it was shown that 2,4,5-TCP and 2,4,6-TCP were present in the urine of adults from 18 to 68 years old in concentrations ranging from 0.1 to 3.8 *μ*g/L and 0.2 to 7.3 *μ*g/L, respectively [42], whereas in urine of adults living in the United States, 2,4,5-TCP and 2,4,6-TCP were determined at concentrations ranging from 3 to 25 *μ*g/L and 3.3 to 65 *μ*g/L, respectively [43]. At the occupational setting, TCP exposure was reported in sawmill workers who were likely exposed to chlorophenols used to prevent fungal growth in lumber after sawing [44]. High concentrations of TCP were also found in blood serum and urine at concentrations ranging from 206 to 1186 *μ*g/L and 196 to 2320 *μ*g/L, respectively, in sawmill worker [44]. The analysis of accumulation of chlorinated phenols in tissues of 58 male and female individuals from Finland who were not occupationally exposed to these substances revealed that both TeCP and PCP were present in adipose tissue and liver at amounts from 2 to 31 *μ*g/kg, whereas TCP was not detected [45]. The previous finding may be connected to the short half life of TCP in tissues (between 1.4 and 1.8 h), which causes much faster elimination of this substance compared with higher chlorinated phenols [46]. 

### 3.3. Effects of Environmental Exposure to Pentachlorophenol and Its Derivatives

Sodium salts of pentachlorophenol (PCP) and tetrachlorophenol (TeCP) have been used extensively as fungicides in the lumber industry since the 1950s. PCP is an environmental toxin that is included in the priority pollutants list of the USA Environmental Protection Agency and the European Union. Although the use of PCP has been strongly limited in the US and other developed countries, it is still employed as a pesticide in wood impregnation in China, and it is commonly exploited in less developed countries [47]. 

Tetrachlorocatechol (TeCC) is one of the main metabolites of PCP. It was observed that TeCC may be formed from PCP in rodents [48]. Moreover, TeCC is one of the main by-products formed during paper production [49]. Tetrachloroguaiacol (TeCG), due to its high potential of accumulation (by 1000-fold) in aquatic biota, may reach concentrations of up to 111 *μ*g/kg of fish [50] and therefore poses serious health risk to consumers of such fish products. The exposure of the population to TeCG may also be related to its presence in drinking water [35] and in the air of areas exposed to industrial pollution [51]. 

### 3.4. Incidence and Effects of Environmental Exposure to Pentachlorophenol and Its Derivatives on Living Organism

Human populations could be exposed to PCP through the migration of this compound from packaging materials (e.g., paper bags) to consumer products with concentration reaching up to 78 *μ*g/kg [52]. PCP concentrations in home dust were observed to be as high as 32 mg of PCP/kg [42], while reports elsewhere [2, 35] documented the presence of PCP in drinking water as a by-product of water disinfection with chlorinated oxidants. Moreover, it was observed that PCP is formed in mammals from pentachlorobenzene (PeCB) [53] and hexachlorobenzene (HCB) [54], chemicals which are commonly used as pesticides and solvents [55]. According to Carrizo et al. [56] children living in the areas of PeCB and HCB emissions were observed to have elevated PCP concentrations in blood serum.

Wagner and colleagues [57] reported residues of PCP found in human testes, kidney, prostate gland, liver, and adipose tissue. PCP is usually found in blood and urine at concentrations ranging from a few to several micrograms [56, 58], PCP concentrations in blood of persons who live in PCP-treated log homes were reported to vary between 69 to 1340 *μ*g/L, while workers permanently exposed to this substance may have PCP amounts of up to 84.9 ppm/L of blood serum [59]. 

Numerous reports have revealed the toxic influence of chlorophenols. It was observed that both PCP and TeCG were powerful uncouplers of oxidative phosphorylation in mitochondria. Moreover, PCP was reported to be promoters of carcinogenesis in rodents [60–62], endocrine disruptors [63], and probable carcinogens in humans [64]. It was also revealed that increased levels of PCP in the blood could lead to severe T-lymphocyte dysfunction [41]. In another study, increased lymphocyte responses were observed in patients with high PCP levels in their blood [65]. According to Brodeur et al. [66], TeCG showed strong toxicity, comparable to that exerted by PCP, whereas Oikari et al. [61] reported that TeCG toxicity was partly related to the inhibition of organic anion transport affecting on the increase of accumulation of other xenobiotics in blood and organs. 

## 4. Ecotoxicity and Health Effects of Chlorophenols

The widespread utilization of chlorophenols for domestic, industrial, forestry, and agriculture purposes has led to their increased burden on the environment [67]. Assessing the environmental risk of chlorophenols in contaminated ecosystems has been an issue of considerable focus, resulting in numerous toxicity tests that utilizes species at variety of organizational levels [67]. Chlorophenol derivatives catechol, chlorocatechols, guaiacol, chloroguaiacols, and syringol exhibit toxic properties including cytotoxic, mutagenic, and cancerogenic activity [68]. Moreover, substitution of these compounds with chlorine atoms may increase their toxicity and prolong the period of bioaccumulation in living organisms [35]. 

### 4.1. DNA Damage in Living Organisms by Chlorophenols

The United States National Report on Human Exposure to Environmental Chemicals in a survey performed between 2002 and 2005 showed the presence of 2,4,5-TCP and PCP in blood serum of individuals [69]. The exposure of a cell to chlorinated compounds usually results in enhanced DNA damage such as double or/and single strand breaks or DNA base oxidation [70]. The teratogenic, neurotoxic, immunosuppressive, cytotoxic, and hepatotoxic effects of 2,4-D have been well documented [71–74]. Other researchers publishing in the open scientific literature have reported oxidant effects of 2,4-D, indicating the potential for cytotoxicity or genotoxicity. For example, Bukowska [75] reported that treatment of human erythrocytes *in vitro* with 2,4-D at 250 and 500 ppm resulted in decreased levels of reduced glutathione, decreased activity of superoxide dismutase, and increased levels of glutathione peroxidase. These significant changes in antioxidant enzyme activities and evidence of oxidative stress indicate that 2,4-D should be taken seriously as a cytotoxic and potentially genotoxic agent. In another study, they noticed that 2,4-DCP and catechol increased the carbonyl group content in human erythrocytes, which was correlated with formation of ROS in these cells [76]. 

Oxidative DNA base damage is mainly related to the formation of highly reactive hydroxyl radical that is produced in the Fenton reaction, in which hydrogen peroxide is converted to hydroxyl radical by transition metal ions such as Fe^2+^ or Cu^2+^ [77]. Bases modifications are repaired primarily by base excision repair [78]. Endonuclease III (Endo III) cut DNA at sites of oxidized pyrimidines provides breaks that can be detected by the alkaline comet assay [79]. Formamidopyrimidine-DNA glycosylase (Fpg) is involved in the first step of the base excision repair to remove specific modified bases from DNA to form an apurinic or apyrimidinic site (AP-site), which is subsequently cleaved by its AP lyase activity giving a gap in the DNA strand [80]. 

Michałowicz and Majsterek [68] analyzed oxidative DNA damage induced by chlorophenols and their derivatives using lesion specific enzymes such as Endo III and Fpg. The use of these enzymes allowed monitoring oxidized pyrimidines and purines by creation of DNA strand breaks at damage sites [81]. The authors also observed DNA damaging effect in samples that were treated with both Endo III and Fpg, which proved that both pyrimidines and purines were oxidized by these xenobiotics. Their findings revealed that the use of Endo III has unveiled more severe DNA damage. Similar results were shown by Andersson and Hellman [82] who observed catechol, induced oxidative DNA damage in human lymphocytes especially in samples treated with this enzyme (Endo III and Fpg). According to the authors, a stronger oxidation of pyrimidines by catechol and/or more efficient repair of catechol-oxidized purines may be responsible for the observation. 

In the study carried out by Michałowicz and Majsterek [68], they observed in their study that chlorocatechols, particularly TeCC, induced more severe damage to DNA bases in comparison to chlorophenols and chloroguaiacols. The authors observed that 2,4,5-TCP and PCP induced oxidation DNA damage. It was also shown that catechols may be oxidized in cells to highly reactive semiquinone radicals [83]. Vatsis and Coon [84] observed that parasubstituted phenols such as 4-chlorophenol were converted to hydroquinone by cytochrome P450 2E1 (CYP2E1), whereas chromosome aberrations and other structural changes within chromosomes were reportedly induced by pentachlorophenol at low concentrations [85]. Damage of DNA was aggravated by the formation of the PCP product, tetrachlorohydroquinone, and harmful intermediate form tetrachlorosemiquinone radical that degraded DNA and handicapped the mechanisms responsible for its repair [86]. Single-cell gel electrophoresis (the comet assay) is a sensitive method for the detection of DNA damage at individual cell levels [68]. It is considered an indicator of genotoxic activity of chemicals in living cells. To date, several authors have used the comet assay to measure xenobiotic-induced DNA damages *in vitro* in human cells [68, 87, 88]. 

### 4.2. Oxidative Stress and Toxicity in Living Organisms

The persistence of chlorophenolic compounds in the environment has resulted in their widespread existence throughout the food chain. Metabolic studies carried out in rodents and human liver homogenates have indicated that PCP undergoes oxidative dechlorination to form tetrachlorohydroquinone (TCHQ) [48]. In the presence of oxygen, superoxide radicals can be produced by the cycle of autoxidation and reduction between TCHQ and its corresponding semiquinone radical under certain physiological conditions [89]. Thus, PCP could present a potent source of reactive oxygen species (ROS) during metabolization.

Free radical catalyzed tissue injury is thought to play a fundamental role in human disease [90]. Particular constraints in addressing this hypothesis have been the inability to assess free radical generation *in vivo* and the lack of information on drugs or vitamins that act as effective antioxidants *in vivo* [91]. Isoprostanes are a family of prostaglandin isomers that are produced from oxidative modification of polyunsaturated fatty acids through a free radical catalyzed mechanism [92]. One of the compounds that can be produced in abundance by such a mechanism is 8-epi-PGF2*α*, a potent vasoconstrictor and a chemically stable end product of lipid peroxidation [93]. Monitoring this compound has been shown to be a useful index of *in vivo* lipid peroxidation [92, 94]. 

Wang and Lin [95] and Wang et al. [96] observed that DNA strand breakage in mammalian cells, glutathione conjugate formation, and the depletion of glutathione content in liver tissue can be induced by TCHQ. In addition, protein adducts and oxidative DNA lesions have also been reported by other investigators [97, 98]. Studies have shown that PCP promotes and initiates liver carcinogenesis, and the promoting effect is related to oxidative stress and compensatory hepatocellular proliferation [98]. Thus, hepatotoxicity generated through oxidative damage is believed to play an important role during the pathophysiological process of liver disease induced by PCP [91]. The investigation led by Bukowska, Duchnowicz, and coworkers revealed numerous toxic effects caused by chlorophenols in human erythrocytes [99–102]. The authors observed that chlorophenols oxidize lipids [100], and proteins [76] and cause reactive oxygen species (ROS) formation [103] and change in antioxidative system (decrease the level of GSH and decreased activity of catalase and superoxidative dismutase [101, 102]). Finally chlorophenols induced changes in erythrocytes morphology (echinocytes formation) and hemolysis of these cells [100–102]. Bukowska et al. suggested that the additional chlorine atom in 2,4,5-TCP is the most probably responsible for high changes in erythrocyte morphology, which may lead to drastic shape changes, that is, to cell shrinkage, hemoglobin leakage, and hemolysis [102]. Additionally, 3-(dimethylamino-)phenol was studied in an other work by Bukowska et al. [76]. It is essential to take into account that prooxidative capability of 2,4-dichlorophenoxyacetic acid is related to 2,4-D hydrolysis to 2,4-DCP that may generate radicals oxidizing [103].

### 4.3. Carcinogenicity of Chlorophenols

The potential carcinogenic effects of chlorophenols were first raised in the 1970s when it was discovered that aquatic and terrestrial milieus might be contaminated with polychlorinated dibenzo dioxins. By the early 1990s, their widespread use as treatment to prevent growth of sapstain fungi on the surface of lumber was discontinued in most countries [104]. The relationship between cancer and exposure to chlorophenols and related chlorophenoxy acid herbicides has been examined in a number of epidemiologic studies. The most consistently observed findings have been excesses of non-Hodgkin's lymphoma [105] and soft tissue sarcoma [106], although excesses of multiple myeloma [107], lung, kidney [28], nasopharyngeal and sinonasal cancers [108] have also been observed. In addition, few studies have provided results specifically for pentachlorophenol or tetrachlorophenol, all with relatively small numbers of exposed people [104]. The evidence regarding the human carcinogenicity of polychlorophenols and their salts was classified by the International Agency for Research on Cancer [109]. Clinical findings have shown that people exposed to chlorophenols fall ill with of tumours, sarcoma, and lung cancer [104]. According to literature findings, the mixture of chlorophenols or sodium salts of these compounds is probably carcinogenic for animals [110]. The U.S. Environmental Protection Agency classified this compound as a carcinogen and the World Health Organization classified catechol in 2B group as a compound of possible carcinogenicity [110]. 

The mechanism of toxicity induced by PCP on mammals and humans has been studied *in vivo *as well as *in vitro*. Tetrachlorohydroquinone (TCHQ), a metabolite of PCP in liver [36], may enhance toxicity and carcinogenicity of PCP, since it is capable of inducing oxidative damage to cellular DNA [98]. *In vitro* studies demonstrated that inhibition of apoptosis induced by PCP in liver and bladder cells contributes to tumor promotion [91, 111, 112]. PCP can induce direct necrosis and its metabolic product 4-chlorohydrocarbohydrate can break DNA chains, producing more severe toxicity than PCP itself [91]. PCP has been proposed to be a promoting agent; Umemura et al. [98] reported the ability of PCP to promote carcinogenesis in mouse livers. During the multistage carcinogenesis, the promotion stage may occur either by growth stimulation of the initiated cell or by prevention of the death of these cells by apoptosis [112, 113]. Gap junctional intercellular communication (GJIC) was thought to be necessary in both processes; both inhibition of GJIC and apoptosis may play a role in tumor promotion [112, 113]. Inhibition of apoptosis was also observed in human bladder cells T-24 and hepatoma cells HepG2 after treatment with PCP [91, 111]. 

The cancer development in people exposed to chlorophenols is related to microsomal activation of cytochrome P450. The oxidation reactions lead to conversion of some xenobiotics to electrophilic forms that actively interacted with cell structures [36]. For instance, pentachlorophenol activation leads to the formation of tetrachloro-1,4-benzoquinone and tetrachloro-1,2-benzoquinone by intermediate steps with formation of respective semiquinone radicals. Formation of tetrachloro-1,4-benzoquinone and tetrachloro-1,2-benzoquinone compounds is also related to liver cancer development in mice. The fundamental is that cancer development is also correlated with the level of microsomal activation of cytochrome P450 of hepatocytes [36]. 

## 5. Biological Monitoring of Chlorophenols in the Environment

The classical procedure used to identify and quantify the chlorophenols includes its extraction and separation from other potentially interfering substances in biological samples; and further quantification by instrumental analysis (GC, LC, MS), genetic toxic assays, enzymatic and bacterial assays, and immunoassays. Although conventional analytical methods offer detection limits in the sub-ppb level, they are labour intensive, require specialized expensive equipment and sometimes suffer recovery losses [114, 115]. Techniques that are not laboratory based (test kits, dipsticks, indicators, portable devices, and real-time monitors) are needed to reduce cost and provide information in time to avoid hazardous chemical exposures. Immunochemical techniques are gaining relevance in the area of human exposure assessment [116]. Immunoassays have been developed for the detection of urinary biomarkers of exposure to pesticides, chlorophenols, and other environmental pollutants, such as triazines, organophosphorus insecticides, carbaryl, naphthalene, and PAHs [117]. 

## 6. Analytical Methods for Detecting Chlorophenols in Environmental and Biological Medium

The techniques used for chlorophenol analysis are quite diverse and depend on the type of matrix sample used. Analytical techniques mainly used in the determination of chlorophenols in environmental and biological samples are gas chromatography with electron-capture (GC-ECD) [118], flame ionization (GC FID), and mass spectrometer (GC-MS) detectors [119]. Liquid chromatography (LC or HPLC) in combination with ultraviolet (UV) radiation [120], electrochemical detection [121], or capillary electrophoresis [122], has also been used. The standard technique for determination of TCPs in water has been reported in the EPA methods 604, 625, and 8041 [123]. They are based on chlorophenol liquid-liquid or solid phase extraction followed by derivatization with diazomethane, methylene chloride or pentafluorobenzyl bromide and GC-FID, GC-ECD, or GC-MS detection. The most frequently employed analytical procedures for chlorophenols involve the use of solvent extraction [124], solid-phase extraction [119], solid-phase microextraction [125], or supercritical fluid extraction [126]. 

## 7. Conclusions and Future Research

Chlorophenols are persistent and recalcitrant toxicants that are widely spread in the environment. The compounds are toxic to aquatic life and have potential to cause histopathological changes, mutagenic, and carcinogenic effects. As analytical methods improve, the detection and quantification of more organic contaminants in the environment become possible. Moving toward a more thorough cataloging of the pollutants present in our ecosystems elucidates the true lifecycle of the synthetic chemicals introduced to the environment. Understanding the crucial outcome of the manufactured chemicals is essential in order to avoid situations analogous to DDT or PCB contamination. Further research needs to be done to determine the potential human and environmental health risks posed by short and long time exposures to mixture of man-made organics in the environment.

## Figures and Tables

**Table 1:** Priority contaminants set by EU and US-EPA.

EU	US-EPA
2-Amino-4-chlorophenol	Phenol
2-Chlorophenol	2-Chlorophenol
3-Chlorophenol	2,4-Dichlorophenol
4-Chlorophenol	4-Chloro-3-methylphenol
4-Chlorophenol-3-methylphenol	2,4,6-Trichlorophenol
2,3,4-Trichlorophenol	Pentachlorophenol
2,4,5-Trichlorophenol	
2,4,6-Trichlorophenol	
3,4,5-Trichlorophenol	
3,5,6-Trichlorophenol	
Pentachlorophenol	
